# Perception of indoor air quality (IAQ) by workers in underground shopping centers in relation to sick-building syndrome (SBS) and store type: a cross-sectional study in Korea

**DOI:** 10.1186/s12889-019-6988-6

**Published:** 2019-05-23

**Authors:** Jeonghoon Kim, Mijung Jang, Kilyoung Choi, KyooSang Kim

**Affiliations:** 1 Department of Environmental Health Research, Seoul Medical Center, 156 Sinnae-ro, Jungnang-gu, Seoul, 02053 Republic of Korea; 2 Department of Chemical Biotechnology, Seokyeong University, 124 Seogyeong-ro Seongbuk-gu, Seoul, 02173 Republic of Korea

**Keywords:** Underground shopping center, Worker, Perception, Sick-building syndrome, Indoor air quality

## Abstract

**Background:**

We examined the indoor air quality (IAQ) perceptions of workers and their relationships with the symptoms of sick-building syndrome (SBS) and store types in underground shopping centers.

**Methods:**

In 2017, 314 store workers in nine underground shopping centers in Seoul, Korea, were assessed. The all participants’ stores were partially opened onto a passage. Using questionnaires, they were asked about their demographics, job characteristics, 16 SBS symptoms, and seven IAQ perceptions. The SBS symptoms were categorized as skin, eye irritation, respiratory, or general. An IAQ perception score was calculated by adding the number of positive responses to each type of IAQ and categorized into three levels.

**Results:**

The prevalence of SBS symptom groups in the previous month was 43.6% for skin symptoms, 62.4% for eye irritation symptoms, 65.6% for respiratory symptoms, and 64.7% for general symptoms. Participants who perceived IAQ were more likely to have SBS symptoms (odds ratio: 1.81–7.84). The type of store employing the workers was associated with several IAQ perceptions. Subjects who worked in clothing and fashion-accessory stores were more likely to have high IAQ perception scores than those who worked in food services.

**Conclusions:**

About half of the store workers in underground shopping centers had experienced SBS symptom groups in the previous month. The SBS symptom groups were associated with almost all IAQ perceptions. Because IAQ perception was associated with store type, studies of IAQ in working areas might need to improve store workers’ health in underground shopping centers.

**Electronic supplementary material:**

The online version of this article (10.1186/s12889-019-6988-6) contains supplementary material, which is available to authorized users.

## Background

As defined by the World Health Organization in 1983 [1], sick-building syndrome (SBS) causes symptoms related to the indoor environment reported by occupants of office buildings. These symptoms include fatigue, headache, and irritation of the upper respiratory tract, eyes, nose, throat, and hand or facial skin [1, 2]. SBS symptoms are associated with personal and environmental factors. Personal factors associated with SBS symptoms include female sex, history of allergies, anxiety, depression, and psychosocial work stress [3–5], while environmental factors include building characteristics (e.g., dampness and poor ventilation) [6, 7], indoor air pollution (e.g., volatile organic compounds (VOCs) and bioaerosols) [8, 9], and perceived indoor air quality (IAQ) such as odor and humidity perception [10, 11]. However, the mechanism by which SBS symptoms are caused is not clear.

Subjective perception of IAQ, including odor perceptions and sensory irritation, is related to indoor air pollutants, such as VOCs and nitrogen oxides [12]. Sources of odors in indoor environments include human body odor, secondhand smoke, bio-odorants (e.g., mold and animal-derived materials), and cosmetics [13]. Sensory irritation of the eyes and airways may be due to airborne compounds stimulating the skin or mucosal tissues, which is often described as “dryness.” Sensory irritation and odor perception are often experienced simultaneously.

Most SBS studies have been conducted in office buildings [9, 14, 15]. However, in our knowledge, studies regarding SBS symptoms of workers in underground spaces in other countries have not been well reported. Worldwide, underground spaces are being used increasingly for various purposes, such as transportation systems and commercial facilities [16]. In metropolitan centers, underground shopping areas are often crowded with shoppers, passengers, workers, and employees.

Although the IAQ in underground shopping centers in Korea is regulated by the Indoor Air Quality Management Act, workers in specific sections or semi-open stores might be exposed to different levels of indoor air pollution due to different emission sources in each store [17]. One study reported that the carbon dioxide (CO), formaldehyde (HCHO), and total volatile organic compound (TVOC) concentrations differed significantly (*P* < 0.05) in different merchandise sections in nine department stores in China [18]. Thus, store employees who spend large amounts of time in stores could be exposed significantly of such pollutants.

In the present study, we investigated the subjective health and IAQ of workers in underground shopping stores. The purposes of this study was to examine the relationships between SBS symptoms and the IAQ perceptions of workers, as well as the associations between IAQ perception and store type in underground shopping areas.

## Methods

### Study site and participants

Seoul, the capital of the Republic of Korea, had 29 underground shopping centers in 2017. We selected nine underground shopping centers based on our preliminary study conducted in March to April (spring season), 2017 [19]. Briefly, several air pollutants (e.g., CO, particulate matter, suspended bacteria, and etc.) were measured in one to three points at the passages in 26 of the 29 underground shopping centers to examine IAQ in these centers. The preliminary study revealed that almost all air pollutants did not exceed the maintenance or recommended standards for Korean IAQ in 2017.

In this study, we focused on prevalence and associated factors of SBS symptoms and perceived IAQ of store workers in underground shopping centers. Based on the preliminary study, we ranked the 26 underground shopping centers according to levels of each air pollutant and scored them to classify three different groups of underground shopping centers by low, middle, and high scores of air pollution [19]. In each group, three underground shopping centers with similar scores were selected.

The selected nine underground shopping centers had a trained interviewer visit stores in each underground shopping center in September (late summer season), 2017. At that time, the nine underground shopping centers had 1143 stores, ranging from 53 to 520 stores per center and their net floor area was more than 3000 m^2^ in each centers [20]. IAQ in these underground shopping centers was controlled by heating, ventilating, and air conditioning (HVAC) system.

In each shopping center, we selected approximately 35 stores ranged from 33 to 36 stores, most of which opened onto a passage. Thus, indoor air might share between stores and passages. The interviewers chose store workers with similar sex and age ratios from across the nine underground shopping centers because we considered sex and age of store workers might be associated with SBS symptoms. However, we did not have any prior knowledge of the SBS symptoms, IAQ perceptions, or other demographic and job characteristics of these workers. One worker in each store was asked to complete a self-administered questionnaire displayed on a tablet computer. In total, 314 workers participated.

### Measurements

The questionnaire included information on demographic and job characteristics, SBS symptoms, and IAQ perceptions (Additional file 1: Table S1). The demographic characteristics included sex, age, education level, and smoking status. The job characteristics included the name of the underground shopping center, length of employment, hours worked per day, floor level, store remodeling status, and store type. We categorized the stores into four different groups with similar characteristics: food service (e.g., restaurants, cafes, and bakeshops), clothing (e.g., clothing stores, clothing repair shops, and bedclothes shops), fashion accessories (e.g., shoe, bag, and briefcase shops), and others (e.g., office, electronic products, and cosmetic shops).

To determine subjective health status, we used 16 SBS questions based on a modified version of the National Institute for Occupational Safety and Health Indoor Air Quality and Work Environment Symptoms Survey (Additional file 1: Table S2). Similar SBS question items have been used in previous studies [14, 21]. The SBS question was “Have you experienced the following symptom while working in the underground shopping center in the last month?” For each SBS symptom, the participants chose one of the following options: “never,” “rarely,” “neutral,” “sometimes,” and “frequently.” We deemed that they had the SBS symptom if they answered “sometimes” or “frequently.” The 16 symptoms were categorized into the following four SBS group based on clinical symptom characteristics: (1) Skin symptoms included “skin dryness or itching.” (2) Eye irritation symptoms included “dry, itching, or irritated eyes” and “tired or strained eyes.” (3) Respiratory symptoms included “stuffy or runny nose,” “cough,” “sneezing,” “sore or dry throat,” “wheezing,” and “shortness of breath or chest tightness.” (4) General symptoms included “nausea or upset stomach,” “headache,” “tiredness, fatigue or drowsiness,” “nervousness,” “difficulty remembering things or concentrating,” “dizziness or lightheadedness,” and “feeling depressed.” Similar groupings of SBS symptoms have been reported [10, 11, 14]. Each symptom group was defined as having symptoms if at least one of the SBS symptoms for that group was reported.

We used perceived IAQ, including odor and humidity perceptions (Additional file 1: Table S3). The odor and humidity perception had been used as qualitative indicators of IAQ in previous studies [10, 11]. The IAQ perception questions were “Have you experienced the following perception while working in the underground shopping center in the last month: (1) stuffy odor; (2) unpleasant odor; (3) pungent odor; (4) moldy odor; (5) tobacco smoke odor; (6) humid air; or (7) dry air?” For each IAQ perception, we used a five-item scale, choosing one of the following options: “never,” “rarely,” “neutral,” “sometimes,” and “frequently.” When a participant answered “sometimes” or “frequently” for each IAQ perception, we considered it a “yes” response (code = 1), while the others were considered a “no” response (code = 0). The IAQ perception score was calculated by adding the number of positive responses for each IAQ and ranged from 0 to 7. Then, the IAQ perception score was categorized into three levels with similar proportions: category 0, score 0 out of 7 “yes” answers; category 1, score 1 or 2 out of 7 “yes” answers; and category 2, score > 2 out of 7 “yes” answers. Similar categorization has been used in a previous study [11].

### Statistical analysis

The chi-square test was used to compare the prevalence of the SBS symptom groups with the workers’ demographic and job characteristics. Because all of the underground shopping centers were located at first-basement levels and only 3% of the stores (9 of 314 stores) had been remodeled within 6 months, these factors were not used as variables. Although the nine underground shopping centers were classified by three different score groups of air pollution, we did not use this variable because the groups of underground shipping centers were not associated with any of SBS symptoms or IAQ perception of store workers (data not shown). Tables S1 and S2 present the relationships between IAQ perception or categorized IAQ perception and the workers’ demographic and job characteristics, respectively (Additional file 2). Spearman correlation was used to assess the relationships among the IAQ perception index. Multivariable logistic regression models were used to examine the relationships between SBS symptom groups and IAQ perception or to determine the relationships between IAQ perception and types of store controlling for workers’ demographics and other job characteristics. We further examined the relationships between SBS symptoms and store types of workers in underground shopping centers using multivariable logistic regression models (Additional file 2: Table S3). Potential variables identified by the chi-square test (*P* < 0.1) were included in the final model. Although several SBS symptoms or IAQ perceptions were not associated with sex, age, smoking status, and store type in the chi-square test, we included these factors in the multivariable logistic models because these variables could affect the observed associations. Because the categorized IAQ perceptions levels were ordinal, the proportional odds assumption in the regression models for IAQ perception was tested using the score test to confirm or reject the assumption. The odds ratio (OR) for the prevalence of SBS symptom groups or IAQ perception of workers was reported with the 95% confidence interval (CI). A *P*-value of 0.05 was deemed significant in all analyses. SAS 9.4 software (SAS Institute, Cary, NC, USA) was used for all statistical analyses.

## Results

The average age of the 314 store workers was 47 ± 13 years and 58% were female. The prevalence of the 16 SBS symptoms in the store workers in the previous month ranged from 12.7 to 57.6% (Table 1). For the SBS symptom groups, the prevalence of SBS symptoms was 43.6% for skin symptoms, 62.4% for eye irritation symptoms, 65.6% for respiratory symptoms, and 64.7% for general symptoms.

**Table 1:** Prevalence of 16 sick-building-syndrome symptoms in store workers in underground shopping centers in the previous month

Group	Symptom	Yes (%)
Skin	Skin dryness or itching	137 (43.6)
Eye irritation	Dry, itching, or irritated eyes	167 (53.2)
	Tired or strained eyes	162 (51.6)
Respiratory	Stuffy or runny nose	123 (39.2)
	Cough	116 (36.9)
	Sneezing	144 (45.9)
	Sore or dry throat	151 (48.1)
	Wheezing	49 (15.6)
	Shortness of breath or chest tightness	52 (16.6)
General	Nausea or upset stomach	40 (12.7)
	Headache	115 (36.6)
	Tiredness, fatigue, or drowsiness	181 (57.6)
	Nervousness	115 (36.6)
	Difficulty in remembering things or in concentrating	90 (28.7)
	Dizziness or lightheadedness	61 (19.4)
	Feeling depressed	40 (12.7)

Table 2 shows the prevalence of SBS symptom groups in the previous month according to demographic and job characteristics. The prevalence of skin, eye irritation, respiratory, and general symptoms differed with several demographic and job characteristics. Particularly, the prevalence of eye irritation and general symptom groups were significantly higher in female workers than in men. Associations SBS symptoms with store type of the workers in the multivariable logistic regression models are presented in Table S3 (Additional file 2).

**Table 2:** Prevalence of sick-building-syndrome symptoms according to the demographic and job characteristics of store workers in underground shopping centers

	Total	Skin		Eye irritation		Respiratory		General	
		Yes (%)	*P*-value	Yes (%)	*P*-value	Yes (%)	*P*-value	Yes (%)	*P*-value
Demographic characteristics									
Sex									
Male	132	52 (39.4)	0.197	68 (51.5)	< 0.001	80 (60.6)	0.112	75 (56.8)	0.013
Female	182	85 (46.7)		128 (70.3)		126 (69.2)		128 (70.3)	
Age (years)									
< 40	91	42 (46.2)	0.022	57 (62.6)	0.102	65 (71.4)	0.013	63 (69.2)	0.078
40–49	81	45 (55.6)		59 (72.8)		61 (75.3)		59 (72.8)	
50–59	90	34 (37.8)		52 (57.8)		53 (58.9)		51 (56.7)	
≥ 60	52	16 (30.8)		28 (53.8)		27 (51.9)		30 (57.7)	
Education									
High school or less	157	66 (42.0)	0.569	95 (60.5)	0.485	98 (62.4)	0.235	100 (63.7)	0.723
College or higher	157	71 (45.2)		101 (64.3)		108 (68.8)		103 (65.6)	
Smoking status									
No	250	106 (42.4)	0.385	156 (62.4)	0.988	160 (64.0)	0.237	157 (62.8)	0.175
Yes	64	31 (48.4)		40 (62.5)		46 (71.9)		46 (71.9)	
Job characteristics									
Length of employment (years)									
< 2	76	31 (40.8)	0.393	51 (67.1)	0.799	51 (67.1)	0.467	53 (69.7)	0.477
2–4	86	44 (51.2)		52 (60.5)		61 (70.9)		58 (67.4)	
5–9	60	26 (43.3)		36 (60.0)		39 (65.0)		35 (58.3)	
≥ 10	92	36 (39.1)		57 (62.0)		55 (59.8)		57 (62.0)	
Hours worked per day (hours)									
< 8	42	12 (28.6)	0.035	22 (52.4)	0.149	27 (64.3)	0.847	21 (50.0)	0.033
≥ 8	272	125 (46.0)		174 (64.0)		179 (65.8)		182 (66.9)	
Type of store									
Food service	28	10 (35.7)	0.159	13 (46.4)	0.337	11 (39.3)	0.017	13 (46.4)	0.086
Clothing	151	64 (42.4)		96 (63.6)		100 (66.2)		96 (63.6)	
Fashion accessories	65	36 (55.4)		42 (64.6)		47 (72.3)		48 (73.8)	
Others	70	27 (38.6)		45 (64.3)		48 (68.6)		46 (65.7)	

Among the store workers, dry air was the most perceived IAQ perception (46.2%), followed by stuffy odor (34.4%), unpleasant odor (28.0%), pungent odor (22.0%), humid air (19.1%), tobacco smoke odor (18.2%), and moldy odor (16.6%). Table 3 shows the cross-correlations between any two IAQ perceptions. All IAQ perceptions were significantly correlated with each other, except for the correlation between humid air and tobacco smoke odor.

**Table 3:** Spearman correlation coefficients among indicators of indoor air quality perceived by workers in underground shopping centers (*n* = 314)

	Stuffy odor	Unpleasant odor	Pungent odor	Moldy odor	Tobacco smoke odor	Dry air	Humid air
Stuffy odor	1.00						
Unpleasant odor	0.67***	1.00					
Pungent odor	0.60***	0.71***	1.00				
Moldy odor	0.42***	0.31***	0.38***	1.00			
Tobacco smoke odor	0.23***	0.28***	0.29***	0.12*	1.00		
Dry air	0.19***	0.13*	0.14*	0.22***	0.18**	1.00	
Humid air	0.14*	0.22***	0.23***	0.15**	0.07	0.23***	1.00

*P < 0.05; **P < 0.01; ***P < 0.001

Each perceived indoor air quality was categorized into no (code = 0) and yes (code = 1)

Table 4 shows the results of the multivariable logistic regression analyses of all four SBS symptom groups for the workers according to IAQ perceptions. After adjusting for demographic and job characteristics, most SBS symptoms were significantly associated with all IAQ perceptions. The ORs of the SBS symptoms for IAQ perceptions ranged from 1.81 to 8.07. Furthermore, the relationships between all of the SBS symptom groups and the categorized IAQ perception score were dose-dependent (*P* for trend < 0.001). Slightly higher effects of IAQ perceptions on SBS symptoms were observed in the multivariable logistic regression analyses without adjustment for store types (Additional file 2: Table S4).

**Table 4:** Relationships between sick-building-syndrome symptoms and indoor air quality (IAQ) perceptions of workers in underground shopping centers

	Skin^a^	Eye irritation^b^	Respiratory^b^	General^a^
	OR (95% CI)	OR (95% CI)	OR (95% CI)	OR (95% CI)
Stuffy odor				
No	1.00	1.00	1.00	1.00
Yes	2.89 (1.73–4.83)***	6.39 (3.40–12.01)***	4.79 (2.56–8.97)***	6.03 (3.12–11.63)***
Unpleasant odor				
No	1.00	1.00	1.00	1.00
Yes	2.08 (1.23–3.51)**	4.06 (2.15–7.67)***	4.00 (2.05–7.81)***	4.29 (2.19–8.41)***
Pungent odor				
No	1.00	1.00	1.00	1.00
Yes	3.07 (1.72–5.50)***	5.16 (2.44–10.89)***	7.33 (3.09–17.4)***	4.46 (2.11–9.42)***
Moldy odor				
No	1.00	1.00	1.00	1.00
Yes	2.29 (1.19–4.41)*	3.65 (1.64–8.13)**	3.81 (1.59–9.09)**	2.93 (1.28–6.71)*
Tobacco smoke odor				
No	1.00	1.00	1.00	1.00
Yes	1.81 (0.99–3.32)	2.45 (1.21–4.99)*	4.04 (1.78–9.13)***	3.29 (1.54–7.02)**
Dry air				
No	1.00	1.00	1.00	1.00
Yes	5.14 (3.07–8.60)***	7.07 (3.99–12.50)***	7.84 (4.28–14.35)***	6.21 (3.47–11.11)***
Humid air				
No	1.00	1.00	1.00	1.00
Yes	2.16 (1.18–3.93)*	2.40 (1.22–4.71)*	3.11 (1.47–6.58)**	2.37 (1.18–4.76)*
Categorized IAQ perception score^c^				
Category 0	1.00	1.00	1.00	1.00
Category 1	5.62 (2.85–11.06)***	4.96 (2.68–9.18)***	5.26 (2.83–9.76)***	3.80 (2.07–6.98)***
Category 2	9.92 (4.91–20.05)***	19.33 (8.75–42.71)***	19.39 (8.47–44.39)***	12.89 (5.96–27.9)***
*p*-for trend	< 0.001	< 0.001	< 0.001	< 0.001

*P < 0.05; **P < 0.01; ***P < 0.001

^a^OR was adjusted for sex, age, smoking status, hours worked per day, and type of store

^b^OR was adjusted for sex, age, smoking status, and type of store

^c^Category 0 = score of 0; Category 1 = score of 1to 2; Category 2 = score of 3 to 7

The relationships between IAQ perception and store type were examined in multivariable logistic analysis adjusting for demographic and job characteristics (Table 5). Participants who worked in clothing (OR = 3.47, 95% CI = 1.10–10.92) and fashion accessory (OR = 4.02, 95% CI = 1.20–13.44) stores were more likely to perceive stuffy odors than those who worked in food services. Participants who worked in clothing (OR = 7.28, 95% CI = 1.64–32.26) and fashion accessory (OR = 5.44, 95% CI = 1.15–25.77) stores were more likely to perceive unpleasant odors than those who worked in food services. Participants who worked in fashion-accessory stores (OR = 13.11, 95% CI = 1.63–105.35) were more likely to perceive a moldy odor than those who worked in food services. Participants who worked in clothing (OR = 2.74, 95% CI = 1.11–6.78) and fashion accessory (OR = 3.46, 95% CI = 1.28–9.35) stores were more likely to perceive dry air than those who worked in food services. Participants who worked in clothing (OR = 2.92, 95% CI = 1.33–6.38) and fashion accessory (OR = 3.25, 95% CI =1.37–7.71) stores were more likely to have high IAQ perception score categories than those who worked in food services. However, pungent odor, tobacco smoke odor, and humid air were not associated with store type.

**Table 5:** Relationships between perceived indoor air quality (IAQ) and type of store for workers in underground shopping centers

IAQ perception	Type of store	OR (95% CI)
Stuffy odor^a^	Food service	1.00
	Clothing	3.47 (1.10–10.92)*
	Fashion accessories	4.02 (1.20–13.44)*
	Others	2.54 (0.75–8.59)
Unpleasant odor^b^	Food service	1.00
	Clothing	7.28 (1.64–32.26)**
	Fashion accessories	5.44 (1.15–25.77)*
	Others	4.64 (0.98–21.94)
Pungent odor^a^	Food service	1.00
	Clothing	1.75 (0.55–5.55)
	Fashion accessories	1.76 (0.51–6.05)
	Others	1.30 (0.37–4.54)
Moldy odor^b^	Food service	1.00
	Clothing	5.32 (0.68–41.65)
	Fashion accessories	13.11 (1.63–105.35)**
	Others	4.29 (0.51–36.03)
Tobacco smoke odor^b^	Food service	1.00
	Clothing	1.60 (0.51–4.96)
	Fashion accessories	1.25 (0.35–4.45)
	Others	1.38 (0.40–4.79)
Dry air^b^	Food service	1.00
	Clothing	2.74 (1.11–6.78)*
	Fashion accessories	3.46 (1.28–9.35)*
	Others	1.97 (0.74–5.24)
Humid air^b^	Food service	1.00
	Clothing	0.40 (0.16–1.03)
	Fashion accessories	0.71 (0.26–2.00)
	Others	0.80 (0.29–2.17)
Categorized IAQ perception score^b,c^	Food service	1.00
	Clothing	2.92 (1.33–6.38)**
	Fashion accessories	3.25 (1.37–7.71)**
	Others	2.05 (0.88–4.78)

*P < 0.05; **P < 0.01

^a^OR was adjusted for sex, age, smoking status, and hours worked per day

^b^OR was adjusted for sex, age, and smoking status

^c^Proportional odds assumption in the cumulative logistic model was met (*P* = 0.78)

## Discussion

Investigation of 16 SBS symptoms for store workers in underground shopping centers revealed that about one to six out of ten workers experienced at least one SBS symptom at their stores in the previous months. The four SBS symptom groups of store workers in underground shopping centers were associated with almost all IAQ perceptions. When IAQ perceptions were dependent variables, several IAQ perceptions were significantly associated with store types of the workers. Particularly, workers in clothing or fashion-accessory stores were more likely to experience several IAQ perceptions than other types of stores. Overall, their effects on IAQ perceptions were higher for workers in fashion-accessory stores than those in clothing stores.

For each SBS symptom group, about half of the workers in stores in underground shopping centers experienced at least one SBS symptom in the previous month. Of the SBS symptom groups, the prevalence of respiratory symptoms was highest. Overall, the percentage of women in all SBS symptom groups was higher than for men, similar to previous studies [3, 11]. This might be because women are more sensitive to SBS symptoms than men [8]. Age, hours worked per day, and store type were associated with several SBS symptom groups.

Workers most often perceived dry air, followed by stuffy odor, unpleasant odor, pungent odor, humid air, tobacco smoke odor, and moldy odor. Most IAQ perceptions were significantly correlated with each other. The correlation was highest between unpleasant and pungent odors. The correlation coefficients between stuffy odor and unpleasant or pungent odors were greater than 0.5, while the other correlation coefficients were less than 0.5. This indicated that different correlation coefficients (low or high) with each other reflected different aspects of the perceived IAQ. Similar findings have been reported when the parents of children reported IAQ perceptions for the home environment in China [22].

Almost all IAQ perceptions were significantly associated with SBS symptom groups in the multivariable logistic analyses. Overall, the IAQ perceptions had higher ORs for respiratory symptoms than for skin, eye irritation, or general symptoms of workers. These trends were consistent with the relationships between SBS symptoms and categorized IAQ perception scores. Dose-dependent relationships were observed between the SBS symptoms and categorized IAQ perception scores. Our findings indicated that IAQ perception might be associated with SBS symptoms of store workers in underground shopping centers. Similar associations have been reported in previous studies. The sensation of dryness among office workers in northern Sweden was associated with SBS symptoms [23]. Perceived stuffy air, dry air, and unpleasant odor were significantly associated with several SBS symptom of workers in public and private companies in Italy [5]. A study conducted in Chongqing, China, reported that IAQ perceptions of parents of children at home were associated with SBS symptoms [10].

Store type was associated with several IAQ perceptions, especially stuffy odor, unpleasant odor, moldy odor, dry air, and categorized IAQ perception scores. Particularly, people who worked in clothing or fashion-accessory stores were more likely to experience several IAQ perceptions and categorized IAQ perception scores than those who worked in food services. Overall, the effects on IAQ perception or categorized IAQ perception score were higher for workers in fashion-accessory stores than those in clothing stores. This suggests that the IAQ may be lower in fashion-accessory stores than in other store types.

The IAQ of stores in underground shopping centers might differ according to the merchandise sold in that store. Fabrics and leather merchandise can contain formaldehyde-based adhesives [24]. The fashion-accessory stores in the present study sold mainly leather products, including shoes, bags, wallets, and backpacks. A study in China reported that when the HCHO and TVOC concentrations were measured in food, clothing, electronic products, leather products, and cosmetics zones in nine underground malls, the HCHO concentrations were generally higher (up to 300 μg/m^3^) in the leather zone than in the other zone types [25]. In that study, the TVOC concentrations were highest in food zones, followed by cosmetic and leather zones. In our study, those who worked in food stores had the lowest ORs on IAQ perception. The food stores we studied included restaurants, cafes, and bakeshops. Although restaurants might have emission sources due to cooking activity, cafes and bakeshops might not have significant emission sources. Therefore, the IAQ perception of the food store workers would not be significantly affected.

This is first study to examined SBS symptoms in store workers in underground shopping areas and their relationships with IAQ perception. We also examined the relationships between the workers’ IAQ perceptions and store type.

This study had several limitations. Because it was cross-sectional, we could not infer that IAQ perceptions were causally associated with the workers’ SBS symptoms. Moreover, because we selected participants in nine underground shopping centers, it might not have been representative of all workers in underground shopping centers in Seoul. Selection of the nine underground shopping centers and 33 to 36 store workers considering sex and age ratios in each center might have biases. Previous studies reported that sex and age was associated with SBS symptoms of workers or occupants in indoor places [3, 8]. Thus, controlling the sex and age ratios across the underground shopping centers might reduce variation of SBS symptoms in different underground shopping centers.

Another limitation was that the participants might have variation of reporting SBS symptoms and IAQ perceptions depending on their sensitivity because we did not use objective methods such as medical examinations by physicians or measurement of IAQ with monitors in each store. Prevalence of SBS symptoms of store workers in underground shopping centers might be excessively estimated because we considered they had SBS symptoms if they answered “sometimes” or “frequently.” Workers with negative disposition might report more SBS symptoms or signs of discomfort for the work environment. Furthermore, recall bias might have affected the prevalence of personal SBS symptoms or IAQ perception. However, the recall bias for SBS symptoms and IAQ perception might not have been a critical issue in our study, since the recall period was short (the previous month).

Although we included several demographic and job factors, there might have been residual confounding of SBS symptoms or IAQ perceptions. Another study reported that personal factors such as a history of allergic disease, anxiety, depression, and psychosocial work stress were risk factors for SBS symptoms [3–5]. Environmental factors, including dampness and ventilation, might also be associated with SBS symptoms or IAQ perception [6, 7]. Unfortunately, we did not measure these factors.

The IAQ in underground shopping centers in Korea is regulated by the Indoor Air Quality Management Act. According to the Act, the IAQ should be measured at a passage. The Act was mainly focused on the shoppers or passengers rather than on store workers. In the Korean Occupational Safety and Health Act, there was limited policy for management of IAQ in such small store in the underground shopping centers. We found that a large percentage of the store workers had SBS symptoms, which were associated with type of stores or IAQ perception. The IAQ perception of the store workers was associated with the types of stores. Further research should investigate IAQ using an objective method at the working-area-level in underground shopping centers.

## Conclusion

A total of 314 store workers in nine underground shopping centers in Seoul participated. About half of these workers had experienced SBS symptom groups in the previous month. The SBS symptom groups were associated with all IAQ perceptions. When the IAQ perceptions were dependent variables, most IAQ perceptions were associated with store type. Those working in fashion-accessory stores were more likely to experience stuffy, unpleasant, and moldy odors, and dry air, and were more likely to have higher categorized IAQ perception scores than those who worked in food services. Our findings suggested that workers in fashion-accessory stores might be exposed to low IAQ.

## Additional files

Additional file 1: **Table S1.** Ten items of demographic and job information. **Table S2.** Sixteen items of sick-building-syndrome symptoms. **Table S3.** Seven items of indoor air quality perception. (DOCX 27 kb)

Additional file 2: **Table S1.** Percentages of indoor air quality perception according to demographic and job characteristics of store workers in underground shopping centers. **Table S2.** Categorized indoor air quality (IAQ) perception scores according to the demographic and job characteristics of store workers in underground shopping centers. **Table S3.** Relationships between sick-building-syndrome (SBS) symptoms and type of store for workers in underground shopping centers. **Table S4.** Relationships between sick-building-syndrome symptoms and indoor air quality (IAQ) perceptions of workers in underground shopping centers without adjustment for type of stores in the multiple logistic regression model. (DOCX 39 kb)

## Abbreviations

CI: Confidence interval
CO: Carbon monoxide
HVAC: Heating, ventilating, and air conditioning
HCHO: Formaldehyde
IAQ: Indoor air quality
OR: Odds ratio
SBS: Sick-building syndrome
TVOC: Total volatile organic compound
VOCs: Volatile organic compounds
