# Supplementary material for: Perception of indoor air quality (IAQ) by workers in underground shopping centers in relation to sick-building syndrome (SBS) and store type: a cross-sectional study in Korea
Source: BMC Public Health. 2019 May 23;19:632. doi: 10.1186/s12889-019-6988-6 (PMC6533716; doi:10.1186/s12889-019-6988-6)
Supplement: Supplementary file 1 — Table S1. Ten items of demographic and job information. Table S2. Sixteen items of sick-building-syndrome symptoms. Table S3. Seven items of indoor air quality perception. (DOCX 27 kb) [file 12889_2019_6988_MOESM1_ESM.docx]

**Additional file 1.**

**Table S1.** Ten items of demographic and job information

| Q1. What is your gender? | 🞏 Male  🞏 Female |
| --- | --- |
| Q2. What is your age now? | __________________ years old |
| Q3. What is the highest level of education you have completed? | 🞏 High school or less  🞏 College or university  🞏 Postgraduate |
| Q4. Are you currently smoking? | 🞏 Never  🞏 In the past but not currently  🞏 Currently smoking |
| Q5. What is the name of underground shopping center? | __________________________ |
| Q6. How long have you worked in your current store? | ______ year (s) ______ month (s) |
| Q7. On average, how many hours did you work a day during the past month? | 🞏 Less than 4 hours  🞏 4 hours or more but less than 8 hours  🞏 8 hours or more but less than 12 hours  🞏 12 hours or more |
| Q8. What floor level are you working at? | __________________________ |
| Q9. Has your store been renovated including remodeling in the last 6 months? | 🞏 No  🞏 Yes |
| Q10. Please check the type of store you work for in underground shopping centers. | 🞏 Shoe store  🞏 Clothing store  🞏 Photo studio  🞏 Restaurants  🞏 Electronic product shop  🞏 Fashion accessories shop  🞏 Cosmetic shop  🞏 Others ( __________________ ) |

**Table S2.** Sixteen items of sick-building-syndrome symptoms

| Q. Have you experienced the following symptom while working in the underground shopping center in the last month? | |
| --- | --- |
| 1) Skin dryness or itching | 🞏 Never  🞏 Rarely  🞏 Neutral  🞏 Sometimes  🞏 Frequently |
| 2) Dry, itching, or irritated eyes | 🞏 Never  🞏 Rarely  🞏 Neutral  🞏 Sometimes  🞏 Frequently |
| 3) Tired or strained eyes | 🞏 Never  🞏 Rarely  🞏 Neutral  🞏 Sometimes  🞏 Frequently |
| 4) Stuffy or runny nose | 🞏 Never  🞏 Rarely  🞏 Neutral  🞏 Sometimes  🞏 Frequently |
| 5) Cough | 🞏 Never  🞏 Rarely  🞏 Neutral  🞏 Sometimes  🞏 Frequently |
| 6) Sneezing | 🞏 Never  🞏 Rarely  🞏 Neutral  🞏 Sometimes  🞏 Frequently |
| 7) Sore or dry throat | 🞏 Never  🞏 Rarely  🞏 Neutral  🞏 Sometimes  🞏 Frequently |
| 8) Wheezing | 🞏 Never  🞏 Rarely  🞏 Neutral  🞏 Sometimes  🞏 Frequently |
| 9) Shortness of breath or chest tightness | 🞏 Never  🞏 Rarely  🞏 Neutral  🞏 Sometimes  🞏 Frequently |
| 10) Nausea or upset stomach | 🞏 Never  🞏 Rarely  🞏 Neutral  🞏 Sometimes  🞏 Frequently |
| 11) Headache | 🞏 Never  🞏 Rarely  🞏 Neutral  🞏 Sometimes  🞏 Frequently |
| 12) Tiredness, fatigue, or drowsiness | 🞏 Never  🞏 Rarely  🞏 Neutral  🞏 Sometimes  🞏 Frequently |
| 13) Nervousness | 🞏 Never  🞏 Rarely  🞏 Neutral  🞏 Sometimes  🞏 Frequently |
| 14) Difficulty in remembering things or in concentrating | 🞏 Never  🞏 Rarely  🞏 Neutral  🞏 Sometimes  🞏 Frequently |
| 15) Dizziness or lightheadedness | 🞏 Never  🞏 Rarely  🞏 Neutral  🞏 Sometimes  🞏 Frequently |
| 16) Feeling depressed | 🞏 Never  🞏 Rarely  🞏 Neutral  🞏 Sometimes  🞏 Frequently |

**Table S3.** Seven items of indoor air quality perception

| Q. Have you experienced the following perception while working in the underground shopping center in the last month? | |
| --- | --- |
| 1) Stuffy odor | 🞏 Never  🞏 Rarely  🞏 Neutral  🞏 Sometimes  🞏 Frequently |
| 2) Unpleasant odor | 🞏 Never  🞏 Rarely  🞏 Neutral  🞏 Sometimes  🞏 Frequently |
| 3) Pungent odor | 🞏 Never  🞏 Rarely  🞏 Neutral  🞏 Sometimes  🞏 Frequently |
| 4) Moldy odor | 🞏 Never  🞏 Rarely  🞏 Neutral  🞏 Sometimes  🞏 Frequently |
| 5) Tobacco smoke odor | 🞏 Never  🞏 Rarely  🞏 Neutral  🞏 Sometimes  🞏 Frequently |
| 6) Humid air | 🞏 Never  🞏 Rarely  🞏 Neutral  🞏 Sometimes  🞏 Frequently |
| 7) Dry air | 🞏 Never  🞏 Rarely  🞏 Neutral  🞏 Sometimes  🞏 Frequently |
