# Supplementary material for: Perception of indoor air quality (IAQ) by workers in underground shopping centers in relation to sick-building syndrome (SBS) and store type: a cross-sectional study in Korea
Source: BMC Public Health. 2019 May 23;19:632. doi: 10.1186/s12889-019-6988-6 (PMC6533716; doi:10.1186/s12889-019-6988-6)
Supplement: Supplementary file 2 — Table S1. Percentages of indoor air quality perception according to demographic and job characteristics of store workers in underground shopping centers. Table S2. Categorized indoor air quality (IAQ) perception scores according to the demographic and job characteristics of store workers in underground shopping centers. Table S3. Relationships between sick-building-syndrome (SBS) symptoms and type of store for workers in underground shopping centers. Table S4. Relationships between sick-building-syndrome symptoms and indoor air quality (IAQ) perceptions of workers in underground shopping centers without adjustment for type of stores in the multiple logistic regression model. (DOCX 39 kb) [file 12889_2019_6988_MOESM2_ESM.docx]

**Additional file 2.**

**Table S1.** Percentages of indoor air quality perception according to demographic and job characteristics of store workers in underground shopping centers

|  | Stuffy odor | | Unpleasant odor | | Pungent odor | | Moldy odor | | Tobacco smoke odor | | Dry air | | Humid air | |
| --- | --- | --- | --- | --- | --- | --- | --- | --- | --- | --- | --- | --- | --- | --- |
|  | Yes (%) | *P*-value | Yes (%) | *P*-value | Yes (%) | *P*-value | Yes (%) | *P*-value | Yes (%) | *P*-value | Yes (%) | *P*-value | Yes (%) | *P*-value |
| **Demographic characteristics** |  |  |  |  |  |  |  |  |  |  |  |  |  |  |
| Sex |  |  |  |  |  |  |  |  |  |  |  |  |  |  |
| Men | 45 (34.1) | 0.923 | 34 (25.8) | 0.446 | 29 (22.0) | 0.999 | 24 (18.2) | 0.510 | 19 (14.4) | 0.141 | 49 (37.1) | 0.006 | 25 (18.9) | 0.948 |
| Women | 63 (34.6) |  | 54 (29.7) |  | 40 (22.0) |  | 28 (15.4) |  | 38 (20.9) |  | 96 (52.7) |  | 35 (19.2) |  |
| Age (years) |  |  |  |  |  |  |  |  |  |  |  |  |  |  |
| <40 | 34 (37.4) | 0.613 | 27 (29.7) | 0.948 | 17 (18.7) | 0.691 | 20 (22.0) | 0.417 | 12 (13.2) | 0.450 | 46 (50.5) | 0.426 | 20 (22.0) | 0.605 |
| 40–49 | 23 (28.4) |  | 21 (25.9) |  | 19 (23.5) |  | 12 (14.8) |  | 18 (22.2) |  | 39 (48.1) |  | 17 (21.0) |  |
| 50–59 | 32 (35.6) |  | 26 (28.9) |  | 19 (21.1) |  | 12 (13.3) |  | 18 (20.0) |  | 41 (45.6) |  | 16 (17.8) |  |
| ≥60 | 19 (36.5) |  | 14 (26.9) |  | 14 (26.9) |  | 8 (15.4) |  | 9 (17.3) |  | 19 (36.5) |  | 7 (13.5) |  |
| Education |  |  |  |  |  |  |  |  |  |  |  |  |  |  |
| High school or less | 52 (33.1) | 0.635 | 43 (27.4) | 0.802 | 33 (21.0) | 0.683 | 28 (17.8) | 0.544 | 26 (16.6) | 0.464 | 76 (48.4) | 0.428 | 28 (17.8) | 0.566 |
| College or higher | 56 (35.7) |  | 45 (28.7) |  | 36 (22.9) |  | 24 (15.3) |  | 31 (19.7) |  | 69 (43.9) |  | 32 (20.4) |  |
| Smoking status |  |  |  |  |  |  |  |  |  |  |  |  |  |  |
| No | 80 (32.0) | 0.077 | 69 (27.6) | 0.740 | 55 (22.0) | 0.983 | 39 (15.6) | 0.366 | 46 (18.4) | 0.822 | 119 (47.6) | 0.318 | 49 (19.6) | 0.661 |
| Yes | 28 (43.8) |  | 19 (29.7) |  | 14 (21.9) |  | 13 (20.3) |  | 11 (17.2) |  | 26 (40.6) |  | 11 (17.2) |  |
| **Job characteristics** |  |  |  |  |  |  |  |  |  |  |  |  |  |  |
| Length of employment (years) |  |  |  |  |  |  |  |  |  |  |  |  |  |  |
| <2 | 26 (34.2) | 0.675 | 21 (27.6) | 0.445 | 15 (19.7) | 0.661 | 11 (14.5) | 0.539 | 9 (11.8) | 0.116 | 30 (39.5) | 0.102 | 16 (21.1) | 0.871 |
| 2–4 | 30 (34.9) |  | 27 (31.4) |  | 19 (22.1) |  | 17 (19.8) |  | 22 (25.6) |  | 44 (51.2) |  | 17 (19.8) |  |
| 5–9 | 17 (28.3) |  | 12 (20.0) |  | 11 (18.3) |  | 7 (11.7) |  | 12 (20.0) |  | 34 (56.7) |  | 12 (20.0) |  |
| ≥10 | 35 (38.0) |  | 28 (30.4) |  | 24 (26.1) |  | 17 (18.5) |  | 14 (15.2) |  | 37 (40.2) |  | 15 (16.3) |  |
| Hours worked per day (hours) |  |  |  |  |  |  |  |  |  |  |  |  |  |  |
| <8 | 9 (21.4) | 0.057 | 8 (19.0) | 0.164 | 7 (16.7) | 0.372 | 3 (7.1) | 0.078 | 10 (23.8) | 0.307 | 19 (45.2) | 0.896 | 9 (21.4) | 0.681 |
| ≥8 | 99 (36.4) |  | 80 (29.4) |  | 62 (22.8) |  | 49 (18.0) |  | 17 (6.3) |  | 126 (46.3) |  | 51 (18.8) |  |
| Type of store |  |  |  |  |  |  |  |  |  |  |  |  |  |  |
| Food service | 4 (14.3) | 0.045 | 2 (7.1) | 0.029 | 4 (14.3) | 0.563 | 1 (3.6) | 0.003 | 4 (14.3) | 0.744 | 8 (28.6) | 0.133 | 8 (28.6) | 0.126 |
| Clothing | 58 (38.4) |  | 51 (33.8) |  | 37 (24.5) |  | 22 (14.6) |  | 31 (20.5) |  | 74 (49.0) |  | 21 (13.9) |  |
| Fashion accessories | 26 (40.0) |  | 18 (27.7) |  | 15 (23.1) |  | 20 (30.8) |  | 10 (15.4) |  | 34 (52.3) |  | 14 (21.5) |  |
| Others | 20 (28.6) |  | 17 (24.3) |  | 13 (18.6) |  | 9 (12.9) |  | 12 (17.1) |  | 29 (41.4) |  | 17 (24.3) |  |

**Table S2.** Categorized indoor air quality (IAQ) perception scores according to the demographic and job characteristics of store workers in underground shopping centers

|  |  | Categorized IAQ score | | |
| --- | --- | --- | --- | --- |
|  | Category 0 | Category 1 | Category 2 | *P*-value |
| **Demographic characteristics** |  |  |  |  |
| Sex |  |  |  |  |
| Men | 49 (37.1) | 44 (33.3) | 39 (29.5) | 0.382 |
| Women | 54 (29.7) | 68 (37.4) | 60 (33.0) |  |
| Age (years) |  |  |  |  |
| <40 | 25 (27.5) | 32 (35.2) | 34 (37.4) | 0.620 |
| 40–49 | 25 (30.9) | 32 (39.5) | 24 (29.6) |  |
| 50–59 | 35 (38.9) | 28 (31.1) | 27 (30.0) |  |
| ≥60 | 18 (34.6) | 20 (38.5) | 14 (26.9) |  |
| Education |  |  |  |  |
| High school or less | 50 (31.8) | 60 (38.2) | 47 (29.9) | 0.634 |
| College or higher | 53 (33.8) | 52 (33.1) | 52 (33.1) |  |
| Smoking status |  |  |  |  |
| No | 82 (32.8) | 91 (36.4) | 77 (30.8) | 0.823 |
| Yes | 21 (32.8) | 21 (32.8) | 22 (34.4) |  |
| **Job characteristics** |  |  |  |  |
| Length of employment (years) |  |  |  |  |
| <2 | 29 (38.2) | 25 (32.9) | 22 (28.9) | 0.381 |
| 2–4 | 27 (31.4) | 29 (33.7) | 30 (34.9) |  |
| 5–9 | 15 (25.0) | 29 (48.3) | 16 (26.7) |  |
| ≥10 | 32 (34.8) | 29 (31.5) | 31 (33.7) |  |
| Hours worked per day (hours) |  |  |  |  |
| <8 | 16 (38.1) | 16 (38.1) | 10 (23.8) | 0.494 |
| ≥8 | 87 (32.0) | 96 (35.3) | 89 (32.7) |  |
| Type of store |  |  |  |  |
| Food service | 14 (50.0) | 10 (35.7) | 4 (14.3) | 0.131 |
| Clothing | 45 (29.8) | 56 (37.1) | 50 (33.1) |  |
| Fashion accessories | 16 (24.6) | 26 (40.0) | 23 (35.4) |  |
| Others | 28 (40.0) | 20 (28.6) | 22 (31.4) |  |

**Table S3.** Relationships between sick-building-syndrome (SBS) symptoms and type of store for workers in underground shopping centers

| SBS symptom | Type of store | OR (95% CI) |
| --- | --- | --- |
| Skin^a^ | Food service | 1.00 |
|  | Clothing | 1.05 (0.43-2.55) |
|  | Fashion accessories | 1.94 (0.74-5.10) |
|  | Miscellaneous | 0.91 (0.35-2.37) |
| Eye irritation^b^ | Food service | 1.00 |
|  | Clothing | 2.24 (0.97-5.21) |
|  | Fashion accessories | 2.62 (1.02-6.73)* |
|  | Miscellaneous | 2.52 (0.99-6.40) |
| Respiratory^b^ | Food service | 1.00 |
|  | Clothing | 3.73 (1.57-8.86)** |
|  | Fashion accessories | 4.45 (1.68-11.79)** |
|  | Miscellaneous | 3.93 (1.52-10.2)** |
| General^a^ | Food service | 1.00 |
|  | Clothing | 1.80 (0.75-4.33) |
|  | Fashion accessories | 3.53 (1.32-9.47)** |
|  | Miscellaneous | 2.30 (0.89-5.97) |

**P*< 0.05; ***P*< 0.01

^a^OR was adjusted for sex, age, smoking status, and hours worked per day

^b^OR was adjusted for sex, age, and smoking status

**Table S4.** Relationships between sick-building-syndrome symptoms and indoor air quality (IAQ) perceptions of workers in underground shopping centers without adjustment for type of stores in the multiple logistic regression model

|  | Skin^a^ |  | Eye irritation^b^ |  | Respiratory^b^ |  | General^a^ |
| --- | --- | --- | --- | --- | --- | --- | --- |
|  | OR (95% CI) |  | OR (95% CI) |  | OR (95% CI) |  | OR (95% CI) |
| Stuffy odor |  |  |  |  |  |  |  |
| No | 1.00 |  | 1.00 |  | 1.00 |  | 1.00 |
| Yes | 2.91 (1.76-4.83)*** |  | 6.62 (3.54-12.39)*** |  | 5.16 (2.77-9.59)*** |  | 6.25 (3.25-11.99)*** |
| Unpleasant odor |  |  |  |  |  |  |  |
| No | 1.00 |  | 1.00 |  | 1.00 |  | 1.00 |
| Yes | 2.04 (1.22-3.42)** |  | 4.22 (2.25-7.92)*** |  | 4.35 (2.25-8.41)*** |  | 4.36 (2.24-8.47)*** |
| Pungent odor |  |  |  |  |  |  |  |
| No | 1.00 |  | 1.00 |  | 1.00 |  | 1.00 |
| Yes | 3.08 (1.73-5.48)*** |  | 5.22 (2.48-10.98)*** |  | 7.26 (3.10-16.98)*** |  | 4.52 (2.15-9.52)*** |
| Moldy odor |  |  |  |  |  |  |  |
| No | 1.00 |  | 1.00 |  | 1.00 |  | 1.00 |
| Yes | 2.54 (1.34-4.82)** |  | 3.80 (1.73-8.31)*** |  | 4.14 (1.77-9.70)** |  | 3.37 (1.49-7.63)** |
| Tobacco smoke odor |  |  |  |  |  |  |  |
| No | 1.00 |  | 1.00 |  | 1.00 |  | 1.00 |
| Yes | 1.77 (0.97-3.24) |  | 2.45 (1.21-4.95)* |  | 3.99 (1.78-8.94)*** |  | 3.36 (1.57-7.20)** |
| Dry air |  |  |  |  |  |  |  |
| No | 1.00 |  | 1.00 |  | 1.00 |  | 1.00 |
| Yes | 5.13 (3.09-8.51)*** |  | 7.12 (4.06-12.5)*** |  | 8.08 (4.46-14.66)*** |  | 6.30 (3.56-11.17)*** |
| Humid air |  |  |  |  |  |  |  |
| No | 1.00 |  | 1.00 |  | 1.00 |  | 1.00 |
| Yes | 2.10 (1.17-3.79)* |  | 2.26 (1.16-4.38)* |  | 2.67 (1.31-5.43)** |  | 2.29 (1.16-4.53)* |
| Categorized IAQ perception score^c^ | |  |  |  |  |  |  |
| Category 0 | 1.00 |  | 1.00 |  | 1.00 |  | 1.00 |
| Category 1 | 5.70 (2.92-11.14)*** |  | 4.78 (2.62-8.74)*** |  | 5.14 (2.82-9.37)*** |  | 3.72 (2.06-6.74)*** |
| Category 2 | 9.86 (4.93-19.74)*** |  | 19.25 (8.82-42.02)*** |  | 20.08 (8.88-45.41)*** |  | 13.31 (6.19-28.60)*** |
| *p*-for trend | <0.001 |  | <0.001 |  | <0.001 |  | <0.001 |

**P*< 0.05; ***P*< 0.01; ****P*< 0.001

^a^OR was adjusted for sex, age, smoking status, and hours worked per day

^b^OR was adjusted for sex, age, and smoking status

^c^Category 0 = score of 0; Category 1 = score of 1to 2; Category 2 = score of 3 to 7
